# Influence of Bisphenol A on Type 2 Diabetes Mellitus

**DOI:** 10.3390/ijerph13100989

**Published:** 2016-10-06

**Authors:** Donatella Paola Provvisiero, Claudia Pivonello, Giovanna Muscogiuri, Mariarosaria Negri, Cristina de Angelis, Chiara Simeoli, Rosario Pivonello, Annamaria Colao

**Affiliations:** 1 I.O.S. & COLEMAN S.r.l., Naples 80100, Italy; donatellapaola.provvisiero@gmail.com (D.P.P.); giovanna.muscogiuri@gmail.com (G.M.); negrimariarosaria@yahoo.it (M.N.); cristinadeangelis83@hotmail.it (C.d.A.); 2 Dipartimento di Medicina Clinica e Chirurgia, Università di Napoli “Federico II”, Naples 80130, Italy; claudia.pivonello@unina.it (C.P.); simeolichiara@gmail.com (C.S.); colao@unina.it (A.C.)

**Keywords:** bisphenol A, diabetes, insulin resistance

## Abstract

Bisphenol A (BPA) is an organic synthetic compound employed to produce plastics and epoxy resins. It is used as a structural component in polycarbonate beverage bottles and as coating for metal surface in food containers and packaging. The adverse effects of BPA on human health are widely disputed. BPA has been recently associated with a wide variety of medical disorders and, in particular, it was identified as potential endocrine-disrupting compound with diabetogenic action. Most of the clinical observational studies in humans reveal a positive link between BPA exposure, evaluated by the measurement of urinary BPA levels, and the risk of developing type 2 diabetes mellitus. Clinical studies on humans and preclinical studies on in vivo, ex vivo, and in vitro models indicate that BPA, mostly at low doses, may have a role in increasing type 2 diabetes mellitus developmental risk, directly acting on pancreatic cells, in which BPA induces the impairment of insulin and glucagon secretion, triggers inhibition of cell growth and apoptosis, and acts on muscle, hepatic, and adipose cell function, triggering an insulin-resistant state. The current review summarizes the available evidences regarding the association between BPA and type 2 diabetes mellitus, focusing on both clinical and preclinical studies.

## 1. Introduction

Type 2 diabetes mellitus (T2DM) is becoming a major public health problem, approaching epidemic proportions [1]. According to the International Federation of Diabetes, in 2015, the global prevalence of diabetes counts 415 million of adult persons, and it is estimated to rapidly increase with a predictable number of 642 million in 2040 [2]. In Italy, over 3.5 million cases of T2DM have been counted in 2015, and it is expected that the number of Italian adult citizens with T2DM will rise to 5.37 million by 2030 [2]. T2DM is a metabolic disease characterized by hyperglycemia, resulting from a progressive loss of insulin secretion on the background of insulin resistance [3]. The pathogenesis of T2DM has been only partially elucidated. Indeed, either genetic or environmental factors contribute to the risk of occurrence of T2DM [4]. In particular, among the environmental factors, mounting evidence suggests that the exposure to environmental endocrine disrupting chemicals (EDCs) might play a role in the pathogenesis of T2DM [5].

Among EDCs, bisphenol A (BPA), which mimics natural endogenous estrogens, has been involved in the pathogenesis of various diseases and, over the last decade, shown to have potential diabetogenic properties. Indeed, several epidemiological studies have suggested that BPA exposure is positively associated with an increased risk of T2DM [6,7,8,9,10,11,12,13]. However, the mechanisms underlying this link between BPA and T2DM are still far from being elucidated.

The current review provides an overview of the available clinical and preclinical studies investigating the potential role of BPA in the pathogenesis of T2DM. The main clinical evidences derive from observational studies reporting an association between BPA exposure, evaluated by the measurement of urinary BPA levels, and the risk of developing T2DM in humans. Moreover, clinical studies on humans and preclinical studies on animals have reported the effects of BPA exposure on the regulation of glucose tolerance, especially during pregnancy or during prenatal and perinatal periods, specific periods in life that are particularly susceptible to BPA action. Finally, a group of preclinical ex vivo studies on human and animal tissues and in vitro studies and animal cell models have investigated the molecular mechanisms of BPA action, focusing on the diabetogenic effect of BPA, mediated by the action on pancreatic Langherans islet cells, involved in insulin production, and on insulin-sensitive peripheral tissues responsible for insulin sensitivity.

### Bisphenol A

BPA is the main component of the clear hard resin polycarbonate; the widespread diffusion of BPA makes the exposure persistent for human beings. Indeed, BPA has been found in many common food containers and packaging, and in the epoxy lining of metal food cans, from which, especially after heating, it can leach into food products [14,15]. The primary source of exposure to BPA for the general population is diet. The exposure different from the dietary intake is lower than dietary exposure by at least one order of magnitude [15]. BPA is a lipophilic synthetic organic compound that, when metabolized, acquires characteristics of hydrophylicity. In humans, after dietary intake, BPA is absorbed through the gastrointestinal tract and transported to the liver [15]. BPA is preferentially metabolized by glucuronidation and sulfation in the microsomal and cytoplasmic fractions of hepatocytes with the formation of glucuronidated and sulfated inactive forms of BPA, which, for their hydrophilic characteristics, are excreted into the bile and, mostly, urine, with a half-life corresponding to about six hours [15,16,17]. In the fetal period, the sulfation is predominant compared to glucuronidation because of a reduced expression of proteins involved in the glucoronidation during pregnancy [16,17]. In rats, BPA administrated orally and via portal vein predominantly undergoes glucuronidation in the liver. The glucuronidated form of BPA is excreted from the liver through the bile into the gastrointestinal tract, where it is metabolically cleaved into unconjugated BPA and glucuronic acid, and the unconjugated BPA is reabsorbed into the blood stream, forming the particular enterohepatic recirculation, which results in the slow elimination of BPA [18,19]. The half-life of BPA in rats after intravenous administration ranges between 30 and 60 min [20]. However, the presence of unconjugated BPA into the blood stream can be due to the presence, in mammalian internal organs including the placenta, of the enzyme β-glucuronidase, which deconjugates BPA, inducing the release of the BPA’s active form [21]. Moreover, due to the lipophilic nature of unconjugated BPA, it gets trapped in adipose tissue from which it is gradually released both in rats and humans [22,23,24].

The BPA parental compound interacts with estrogen receptors (ERs); therefore, it may have a major role during specific periods in life, especially in women, during pregnancy and lactation. Moreover, BPA results in gender-specific clinical effects, including ovarian and uterine dysfunction in females and sexual dysfunction in males [25]. Conversely, metabolites of BPA do not bind to or activate the ER [26]. Furthermore, the differences between males and females mainly involve BPA metabolism. Indeed, glucuronidation and sulfation are generally faster in men than in women [27]. Gender differences have been found in serum BPA concentrations, probably due to the capability of androgens to inhibit the enzyme involved in the process of glucuronidation. Indeed, male subjects, compared with female subjects (both fetuses and adults), have shown higher serum BPA levels when exposed to equal levels of BPA. Moreover, women with hyperandrogenism showed higher serum BPA levels than normal women [28]. Furthermore, gender differences in BPA actions might depend on the urinary excretion and storage of BPA in internal organs. Females have proportionally more body fat than males [29]; hence, they may store relatively more BPA. In addition, males have proportionally higher rates of renal clearance [30]; therefore, females may retain a higher proportion of BPA, even when it has been metabolized.

According to a World Health Organization (WHO) report, the assessment of daily human exposure to BPA, evaluated by measuring the urinary excretion of BPA metabolites, and the daily dietary intake, evaluated by measuring BPA concentrations in food, may vary widely. On the basis of these evidences, in Europe, it has been estimated that BPA daily intake for babies is about 0.2 μg/kg in breast-fed babies and about 11 μg/kg in formula-fed babies using polycarbonate bottles. The estimated daily intake for adults is about 1.5 μg/kg. Moreover, WHO, the Food and Drug Administration (FDA), and the European Food Safety Authority (EFSA) have determined that, regarding dietary exposure to BPA, the no-observed-adverse-effect-level (NOAEL) for the systemic toxicity of BPA is 5 mg/kg body weight/day [31].

## 2. Epidemiological Evidences: Observational Studies in Humans

In this last decade, a number of studies have investigated the association between BPA and T2DM, mostly on the basis of the results derived from data of the National Health and Nutrition Examination Survey (NHANES) [5,6,7,8,9,10,11,32,33]. Lang et al. found a positive association between urinary BPA levels and prevalence of T2DM using cross-sectional data from NHANES 2003–2004 [6]. Melzer et al. failed to demonstrate any associations in the NHANES adult population collected in 2005–2006, probably because the geometric mean value (1.79 ng/mL) of urinary BPA established in the 2005–2006 population was around 30% lower than the geometric mean value (2.49 ng/mL) of urinary BPA established in the 2003–2004 population; however, pooling together data of the two populations lead to a persistence of the association [34]. In 2011, Shankar et al. analyzed samples collected by NHANES between 2003 and 2008 using a criterion for defining diabetes—instead of the self-reported diagnosis as in the previous two studies—fasting glucose or glycosylated hemoglobin levels, as recommended by American Diabetes Association guidelines [8]. In this study, high urinary BPA levels (>4.20 ng/mL) were found to be associated with the development of T2DM independently of traditional diabetes risk factors [8]. Silver et al. reported a correlation between urinary BPA and glycosylated hemoglobin levels in the pooled analysis of 2003–2004, 2005–2006, and 2007–2008 data [9]. LaKind et al. analyzed four different datasets coming from NHANES (2003–2004, 2005–2006, 2007–2008, and 2009–2010) using scientifically and clinically supportable exclusion criteria and outcome definitions, failing to find any association between urinary BPA content and T2DM, concluding that the use of cross-sectional datasets such as NHANES was inappropriate for drawing conclusions between BPA exposure and a complex disease such as T2DM [10]. Nevertheless, the association between urinary BPA levels and T2DM risk, independently of traditional diabetes risk factors, was confirmed in studies considering adult populations different from those of NHANES [32,33]. Notably, the association of BPA urinary levels with T2DM risk seems to be influenced by genetic predisposition, such as single nucleotide polymorphysms in obesity-associated genes, which makes the subjects more sensitive to the deleterious effect of BPA [11]. It is noteworthy that most of the above mentioned studies had the main limitation of being cross-sectional studies, with the inability to determine the temporal sequence of exposure and outcome, and the inclusion of confounding factors affecting the relationship between the putative cause and the assessed effect. Furthermore, the majority of these studies are population surveys or pharmacovigilance studies that are not designed to address the effect of BPA on T2DM. Finally, the single urinary measurement of BPA performed in most studies cannot provide suitable information on long-term exposure. In summary, the limitations related to study design and exposure assessment cannot permit definitive conclusions on the effect of BPA on T2DM. Longitudinal studies investigating the relationship between repeated measures of BPA exposure and repeated health follow-ups might provide insightful evidence on the diabetogenic effect of BPA.

## 3. Clinical Studies on Humans and Preclinical Studies on Animals: BPA Effect on Glucose Tolerance

Several studies support the BPA detrimental effect on pancreatic β cells, with the consequent impairment of insulin secretion and glucose metabolism, but also suggest an “obesogen” action of BPA by affecting adipocyte metabolic functions, with the consequent development of insulin resistance. Insulin resistance is a pathological state characterized by an impairment of insulin action in different compartments, including adipose tissue [35]. The diabetogenic action of BPA is mostly pronounced when humans and animals are in stages of rapid growth, as demonstrated by several studies showing that BPA induces metabolic disorders in exposed human and animal models during pregnancy as well as prenatal and perinatal periods for animals and fetal periods and childhood in humans [36,37,38,39,40,41,42,43,44,45,46].

The effect of BPA on glucose tolerance has also assessed some clinical human studies, which have mainly reported gender-related effects on adipose tissue metabolism in children of women exposed to BPA during pregnancy. In a Taiwan study, male newborns of women exposed to the highest quartile BPA levels during pregnancy had higher leptin levels than female newborns; the mean of maternal and fetal BPA levels were 2.5 ng/mL and 0.5 ng/mL, respectively [36]. In a Mexican-American study, a cohort of women was enrolled during pregnancy, and urinary BPA levels were measured at early (13 weeks) and late (26 weeks) pregnancy. In nine-year-old offspring, adiponectin and leptin serum levels were evaluated and associated with urinary BPA levels of their mothers. Late pregnancy urinary BPA concentrations were positively associated with high leptin levels in nine-year-old boys. Additionally, early pregnancy urinary BPA concentrations were positively related to adiponectin levels in nine-year-old girls [37]. In a Canadian study, adiponectin and leptin levels in male and female infants were measured and correlated with BPA exposure during the first trimester of pregnancy of their mothers; an inverse correlation was found between maternal urinary BPA and adiponectin levels in males, and higher leptin levels were reported in female infants compared with male infants [38]. In summary, the BPA exposure of women during pregnancy induces higher leptin levels in newborns and infants, with gender differences based on BPA exposure during early or late pregnancy.

The effect of BPA on glucose tolerance has also been assessed in preclinical animal studies. Ryan and co-authors treated pregnant mice with BPA by dietary administration at the dose of 0.25 μg/kg/day for the entire duration of pregnancy, estimated to be in the range of daily human adult dietary exposure in the United States, and found an early (4 weeks of age) accelerated growth in the offspring without any impairment of glucose tolerance in 15-week-old mice [39]. In the same period, Alonso-Magdalena et al. treated pregnant mice with BPA by subcutaneous administration at a dose of 10 or 100 μg/kg/day during days 9–16 of gestation; low doses (10 μg/kg/day), but not high doses (100 μg/kg/day), of BPA affected the metabolic status of the mothers, with impairment of glucose tolerance and increased insulin and leptin levels, as well as an impairment of lipid profiles, both during and after pregnancy, until four months after delivery. The same study reported a transgenerational effect of BPA; indeed, the six-month-old male offspring of mothers with insulin resistance developed insulin resistance and an impairment of glucose tolerance, associated with higher levels of plasma insulin compared with offspring of untreated mothers [40]. Although the impairment of glucose metabolism registered in offspring was reported to be a consequence of the BPA effect on the mothers’ metabolisms, the evidence that BPA is able to cross the placental barrier cannot permit the exclusion of the possibility of a direct effect of BPA on offspring metabolism. The discrepancies between these two studies of Ryan and Alonso-Magdalena [39,40] could be due to different experimental settings, the different times of evaluation of the metabolic states in offsprings, and the different BPA dosages.

In a study by Angle et al., pregnant mice were fed daily from gestational day 9 to 18 with a chow diet and the administration of BPA at doses of 5, 50, 500, 5000, and 50,000 μg/kg/day. The wide BPA dose range used in this study extends from 10-fold below the currently estimated reference dose (50 μg/kg/day) to 10-fold above the estimated NOAEL (5000 μg/kg/day). The prenatal exposure of fetuses, through the exposition of pregnant mice at doses of BPA (5, 50, 500, 5000 μg/kg/day), induced postnatal body weight gain, impairment of glucose tolerance and insulin resistance with hyperinsulinemia, an increase in adipocyte number and volume with consequent increase in abdominal fat, and an increase in adiponectin and leptin levels mainly in the group of male fetus treated with 500 μg/kg/day. Notably, while all of these outcomes occurred at doses of BPA at or below the estimated NOAEL, none of these outcomes was statistically different from the negative controls for the highest dose (50,000 μg/kg/day) of BPA that was administered [41].

Additional studies have confirmed that pregnant mice exposed to low doses (10 or 40 μg/kg/day) of BPA during pregnancy and lactation produce offspring that, although fed with a normal diet, can develop insulin resistance with compensatory hyperinsulinemia and impairment of glucose tolerance in adult age, suggesting that BPA can mimic the effects of a high-fat diet [43,44,47,48]; a high-fat diet is also able to accelerate and worsen the detrimental effect of BPA [44,47,48]. Conversely, prenatal exposure to high doses (250 and 1250 μg/kg/day) of BPA did not show any impact on glucose tolerance or insulin sensitivity in the offspring fed with normal or fat diet [47].

Alonso-Magdalena et al. demonstrated that female mice exposed to BPA, administered subcutaneously on days 9–16 of gestation, exhibited impairment of glucose tolerance even 7 months after delivery, with a decreased pancreatic β cell function and β cell mass, possibly a consequence of a prolonged insulin resistance [45]. Notably, the exposure to BPA induced the impairment of glucose tolerance in pregnant mice only when the exposure occurred during gestation or lactation, as confirmed by the lack of effects in non-pregnant female mice at six months by the end of treatment with the same doses of BPA [45]. Moreover, the time of the maternal exposure to BPA seems to have a predominant role not only for the metabolic consequences in the mothers but also for the metabolic consequences in the offspring, which receive the worst metabolic dysfunction, with insulin resistance and β cell dysfunction, and persistent impairment in glucose tolerance when exposed during fetal life [42].

The impairment of glucose metabolism in offspring of mice exposed to BPA during pregnancy can be due to a direct effect of BPA in inducing epigenetic alterations of the *glucokinase* (*Gck*) promoter gene in the hepatic methylome of offspring [43,49,50]. As a consequence of *Gck* methylation, *glucose-6-phosphate dehydrogenase* (*G6p*) messenger levels decreased in the hepatic tissue of BPA-treated offspring compared with the controls, contributing to the subsequent pathogenesis of glucose intolerance [49].

In summary, preclinical animal studies demonstrate that low levels of BPA exposure during pregnancy and lactation can affect glucose tolerance in pregnant mice but also in the offsprings. On the latter, the effect can be direct through epigenetic alteration or indirect through metabolic dysfunction of the mothers. This metabolic effect can be worsened by a fat diet.

Beyond rats, different animal models have been used to test the effect of BPA on glucose tolerance. In sheep, prenatal BPA exposure, at low (0.05 mg/kg/day), medium (0.5 mg/kg/day), and high (5 mg/kg/day) concentrations, leads to metabolic disorders, including insulin resistance, an increase in adipocyte size, and an increase in macrophages in adipose tissue not influenced by postnatal overfeeding [51].

## 4. Preclinical Animal Studies: Molecular Mechanisms of Action of BPA

In vitro and ex vivo animal studies have hypothesized different mechanisms of BPA action as a causative factor of T2DM [52,53,54,55]. Current evidences show that BPA exerts its primary endocrine disrupting activity by interacting with nuclear ER and consequently inducing modifications in estrogen-responsive gene expression [52,56,57]. Both ER isoforms—ER alpha (ERα) and beta (ERβ)—are present in the pancreas, muscle, liver, and adipose tissues [53]. Estrogens binding to ER at concentrations above or below physiological range, can be detrimental for pancreatic Langherans islet cell (particularly for β cells) function and insulin-sensitive peripheral tissues, impairing insulin secretion and insulin sensitivity, and may promote T2DM [58]. The BPA binding affinity to ER is extremely weak—1000 to 10,000 times lower than the ER natural ligand 17β-estradiol—thus suggesting that BPA might have additional mechanisms of action, which are independent of ER [15,59]. The majority of studies reporting the mechanisms of action of BPA on pancreatic Langherans islets have focused on β cells, but data on α cells have been also described. Conversely, no data concerning the effects of BPA on δ, ε, and γ cells of Langerhans islets are available. A different group of studies have described BPA mechanisms of action on the insulin-sensitive peripheral tissues involved in the development of insulin resistance, including adipose tissue, skeletal muscle, and the liver.

### 4.1. BPA Action on Pancreatic β Cells: Cell Studies

The studies investigating the effect of BPA on pancreatic β cells are not homogeneous in terms of BPA concentrations used in the experimental setting and time of BPA exposure, which can be summarized in three different categories: short-term (minutes–hours), long-term (48–72 h), and chronic (weeks) treatment.

In the rat insulinoma cell line (INS-1 832/13), BPA (10 and 100 nM) increased basal insulin secretion after short-term treatment (2 h) [60]. In a mouse β cell line (TC-6), BPA (100 ng/mL) enhanced glucose stimulated insulin secretion after short-term treatment (1 h) and increased the endoplasmic reticulum stress, but did not exert any effects on mitochondrial function after 24 h [61]. In pancreatic β cells within intact isolated Langerhans islets from adult male rats, 24 h of treatment with BPA (0.5 and 25 μg/L) induced a transformation of mitochondria, with a loss of structural integrity and increased β-cell mass [62]. In pancreatic β cells within intact Langerhans islets isolated through digestion with collagenase from adult male mice, low doses (1 and 10 nM) of BPA increases insulin gene expression, insulin synthesis, and insulin secretion after long-term treatment (48 h) through a mechanism involving the activation of ERα [53]. Moreover, the administration of low doses (0.0020 μM) of BPA in the rat insulinoma cell line (INS-1) decreases cell viability, enhances glucose-stimulated insulin secretion, insulin expression, and insulin content, whereas high BPA doses (0.20 and 2 μM) reduce glucose-stimulated insulin secretion and decrease the expression of genes involved in glucose-stimulated insulin secretion, such as *glucose transporter 2* (*GLUT2*) and *Gck*, after long-term treatment (48 h) [63]. It is noteworthy that the insulin hypersecretion after BPA treatment is associated with the induction of mitochondrial stress and activation of B-cell lymphoma 2 (Bcl-2) family members and caspases, responsible for apoptosis of pancreatic β cells [63]. Chronic exposure to BPA (12 weeks) did not affect the pancreatic insulin content, nor the β cell structure or function [64].

The short-term effect (2–8 min) of low doses (1 nM) of BPA in pancreatic β cells is mainly due to the rapid enhance of the frequency of glucose-induced Ca^2+^ ions oscillations, mimicking the effect of 17β-estradiol through a non-classical membrane ER (ncmER) involved in the non-genomic actions of estrogens and xenoestrogens, unrelated to both ERα and ERβ [65]. Moreover, the short-term (5 min) administration of low doses (1 nM) of BPA in Langerhans islets derived from adult male mice induces, via ncmER, an increase in the activation of the ubiquitous transcription factor *cAMP response element binding protein* (*CREB*) [66], which is involved in insulin gene expression [67] and pancreatic β cell survival [68]. Moreover, immunohistochemical data, performed on the pancreatic β cell area of Langherans islets derived from female mice seven months after delivery, showed that chronic exposure to 10 and 100 μg/kg/day of BPA during pregnancy induces the increase in *p16* and *p53* and the decrease in *cyclin D2*, the master regulators of the cell cycle, inhibiting β cell growth and proliferation [45].

BPA has been hypothesized to induce pancreatic β-cell damage also acting on human islet amyloid polypeptide (hIAPP), a soluble monomer of 37 residues, synthesized and secreted by the pancreatic β cells and involved in glycemic regulation promoting satiety. The hIAPP can misfold in toxic oligomers and form cytotoxic fibrils inducing membrane damage of the pancreatic β cells [54,55]. Although the underlying mechanism has not been clearly established, BPA has been demonstrated to promote the aggregation of hIAPP in fibrils, with a consequent disruption of β cell membranes in the rat pancreatic INS-1 cell line [69]. This direct effect of BPA on amyloid fibril formation has also been demonstrated to be associated with the generation of reactive oxygen species, responsible for pancreatic β cell apoptosis [69]. The β cell disruption or apoptosis may mediate the BPA-induced promotion of T2DM. In summary, contrary to the diabetogenic effect, short- and long-term BPA exposure increases insulin secretion in pancreatic β cells. This insulin hyperproduction could, in pathophysiological conditions, balance the insulin resistance in peripheral tissues. Nevertheless, BPA exposure is associated with mitochondrial and endoplasmic reticulum stress. Both events lead to β cells dysfunction and cell apoptosis.

### 4.2. BPA Action on Pancreatic α Cells: Cell Studies

One single study has investigated the effect of BPA on pancreatic Langherans islet α cells, hypothesizing a role of these cells in the development of T2DM induced by BPA. In α cells within intact Langerhans islets from adult male mice, with short-term treatment (2–8 min) with low doses (1 nM), BPA reduced the typical pattern of low glucose-induced intracellular Ca^2+^ ion oscillations, responsible for a decrease in glucagon release and a consequent dysregulation of glucose metabolism [70]. This effect, similar to pancreatic β cells, is very likely triggered by the activation of ncmER; the binding of BPA to ncmER induces nitric oxide synthase (NOS) activation, with the production of nitric oxide, which activates a guanylate cyclase, responsible for the increase in guanosine 3′, 5′-cyclic monophosphate (cGMP) and the downstream activation of the cGMP-dependent protein kinase (PKG) that blocks ion channels [70].

### 4.3. BPA Action on Insulin Sensitivity in Peripheral Tissues: Human, Animal and Cell Studies

The effect of BPA on insulin sensitivity in peripheral tissues, including adipose tissue, skeletal muscle, and the liver, has been assessed on in vivo rodent models and on primary cultures of ex vivo tissues from humans and rodent models, as well as on in vitro rodent cell lines.

In the in vivo studies, adult male mice exposed to BPA at the dose of 10 or 100 μg/kg for a chronic treatment of 4, 8, and 15 days and for 12 weeks developed hyperinsulinemia, insulin resistance, and an impairment of glucose tolerance [52,64,71,72,73]. Moreover, BPA decreased adiponectin levels and increased circulating levels of adipocytokines, such as interleukin-6 and tumor necrosis factor, favoring the development of insulin resistance in adult male mice [64]. Reduction of adiponectin secretion after short-term (6 h) low-dose (1 nM) BPA treatments is also supported by an ex vivo study on human adipose explants obtained from breast reductions, abdominoplasty, and gastric bypass surgery [74].

The development of insulin resistance and impairment of glucose tolerance in mice treated with BPA finds an explanation in the deregulation of insulin receptor pathway in mice skeletal muscles and livers. Indeed, ex vivo studies of BPA-treated mice and adult male albino rat skeletal muscles demonstrated a significant decrement of protein kinase B (Akt) and glycogen synthase kinase 3 beta (GSK3β) phosphorylation, which contributes to the development of an insulin resistance state and glucose intolerance [64,71,73]. In particular, male albino rats treated for 30 days with 20 mg/kg/day and 200 mg/kg/day of BPA showed significant decreases in *glucose transporter 4* (*GLUT4*) expression and glucose oxidation in skeletal muscles [73], whereas mice treated over a long period (8 days) with a low dose of BPA (100 μg/kg) showed significant decreases in the phosphorylation of insulin receptor β subunit upon insulin administration in the liver with consequent impairment insulin signaling [71]. Moreover, BPA exposure at low (20 mg/kg body weight) and high (200 mg/kg body weight) doses induces increased pancreatic β cell insulin release in blood stream and impairs hepatic glucose oxidation and glycogen content through defective insulin signal transduction, such as reduction in Akt phosphorylation, in adult male albino rats [75]. In the same study, GLUT2 protein unexpectedly increased in the livers of male albino rats treated with BPA, possibly due to the ability of BPA to upregulate messenger expression of *peroxisome proliferator-activated receptor gamma* (*PPARγ*), whose response element (PPRE) is present on rat *GLUT2* genes [75].

The development of insulin resistance and impairment of glucose tolerance in mice treated with BPA may be mediated by the BPA action on adipose tissue. The reduced adiponectin release after short-term treatment (24 h) of BPA at increasing doses (20–80 μM) has been found in the 3T3-L1 mouse adipocyte cell line. This effect was associated with a lower activation of Akt and a deregulation of the intracellular insulin receptor pathway [76]. Conversely, in a different clone of the mouse adipocyte cell line (3T3-F442A) BPA (1 μM and 100 μM) increased basal and insulin-stimulated glucose transport with a consequentially increased amount of GLUT4 [77]. In partial agreement with these data, another study showed that, following long-term (24 h and 48 h) BPA treatment (1 nM and 100 nM), basal glucose utilization tended to increase in the 3T3-L1 adipocyte cell line, while insulin-stimulated glucose utilization was downregulated in the 3T3-L1 adipocyte cell line as well as in primary cultures of differentiated adipocytes. The increment in basal glucose utilization was accompanied by increased levels of glucose transporter 1 (GLUT1), but without any change in GLUT4 expression after 24 and 48 h of treatment [78]. Conversely, in another study, BPA treatment has been shown not to increase glucose uptake at doses ranging from 1 fM to 1 μM, in 3T3-L1 adipocytes [79]. The reduced release of adiponectin by adipocytes, together with a deregulation of insulin intracellular pathway in adipocytes, may contribute in the development of insulin resistance and impairment of glucose metabolism induced by BPA exposure. In summary, in vivo, ex vivo, and in vitro studies demonstrate that BPA exposure induces decreased adiponectin production by adipose cells and deregulates insulin receptor signaling and GLUT4 expression in muscles and livers, inducing insulin resistance.

Furthermore, BPA exposure (100 g/kg/day) perturbed the insulin signaling and glucose transport in the mice brain as well, determining a brain insulin resistance state [72]. Indeed, in brain explants, BPA induced an increase in insulin levels in both hippocampus and prefrontal cortex, but decreased insulin receptor expression, insulin receptor substrate 1 (IRS1), AKT, GSK3β, and extracellular signal-regulated kinases (ERK) phosphorylation, implicated in the glucose transport and consequently in the development of brain insulin resistance [72].

## 5. Conclusions

The data summarized in the current review point out that several observational studies have reported an association between BPA exposure and an increased risk of developing T2DM. In addition, preclinical animal studies confirm that exposure to low doses (but not high doses) of BPA during pregnancy and lactation induces the impairment of glucose tolerance and an insulin resistance state in pregnant mice and their offspring. These data are supported by preclinical human and animal ex vivo and in vitro studies from which it was found that BPA, via non-genomic actions, through ncmER, when administered in short-term treatment, and genomic actions, through ER, when administered in long-term treatment, exerts its effects on the pancreas and peripheral tissues. Despite the increased insulin secretion, BPA administration results in mitochondrial and endoplasmic reticulum stress in pancreatic β cells, leading to the deregulation of β cell function and to cell death. Moreover, BPA affects the secretion of adiponectin, inducing a decreased secretion by adipocyte, and induces the impairment of insulin receptors signaling in skeletal muscle and liver, contributing to the development of insulin resistance. However, the interventional studies, concerning the relationship between BPA and T2DM, provide conflicting results most likely due to differences in experimental designs, the wide range of BPA doses applied, the time of exposure, and uncontrolled or residual confounding factors, such as the route of the administration of BPA.
